# Revisional Surgical Options After Single Anastomosis Sleeve Jejunal Bypass for Refractory Biliary Reflux: A Prospective Comparative Study Between Roux-en-Y Gastric Bypass and Reversal to Sleeve Gastrectomy

**DOI:** 10.1007/s11695-026-08772-5

**Published:** 2026-06-11

**Authors:** Mohamed A. Elshimy, Ahmed M. Farrag, Gamal M. Abdalla

**Affiliations:** Ain Shams University, Cairo, Egypt

**Keywords:** Biliary Reflux, Single Anastomosis Sleeve Jejunal Bypass, Revisional Bariatric Surgery, Roux-en-Y Gastric Bypass, Sleeve Gastrectomy, Bile Reflux Management, Gastroesophageal Reflux Disease, Metabolic Outcomes

## Abstract

**Background:**

After Single Anastomosis Sleeve Jejunal (SASJ) bypass, biliary reflux is a challenging postoperative issue that may require revisional intervention. Conversion to Roux-en-Y gastric bypass (RYGB) and revision to sleeve gastrectomy are two frequently used techniques. Their clinical results are assessed and contrasted in this study.

**Materials and Methods:**

Forty patients were the subject of a prospective comparative analysis presenting with persistent biliary reflux after SASJ despite medical therapy. Patients were managed by either conversion to RYGB (Group A, *n* = 22) or revision to sleeve gastrectomy (Group B, *n* = 18). Reflux resolution, gastroesophageal reflux disease (GERD), perioperative outcomes, and weight changes were among the outcomes evaluated over a 12-month period.

**Results:**

The two groups’ baseline characteristics were comparable (*p* > 0.05). The RYGB group had significantly longer operating times and longer hospital stays (*p* < 0.001 and *p* = 0.036). While 27.8% of patients in the sleeve group had persistent reflux, all patients undergoing RYGB showed complete resolution of biliary reflux (*p* = 0.008). GERD outcomes improved in Group A but significantly worsened in Group B (*p* = 0.007). Weight evolution favored RYGB, demonstrating sustained BMI reduction, while sleeve revision was associated with progressive weight regain.

**Conclusion:**

RYGB conversion was associated with more reliable reflux resolution and better overall clinical outcomes compared with sleeve revision. To validate these results, more extensive research is needed.

## Introduction

With a proven link to metabolic conditions like type II diabetes mellitus, hypertension, dyslipidemia, and obstructive sleep apnea, which all raise morbidity and mortality, obesity has emerged as a major global health concern [1, 2].

The most effective strategy for attaining long-term weight loss and enhancing metabolic profiles has been shown to be metabolic and bariatric surgery. Reduced operating time and preserved endoscopic access are two advantages of the Single Anastomosis Sleeve Ileal (SASI) bypass, which combines a restrictive sleeve component with a loop intestinal bypass via a single anastomosis [3, 4].

To reduce nutritional issues while maintaining metabolic efficacy, a modified version known as the Single Anastomosis Sleeve Jejunal (SASJ) bypass was developed using a shorter biliopancreatic limb [5]. Biliary reflux has become a clinically significant postoperative concern despite these benefits. Reflux symptoms and mucosal damage can result from the loop configuration’s retrograde passage of pancreatic and biliary secretions into the stomach lumen and possibly the esophagus [6, 7].

**Fig. 1:**
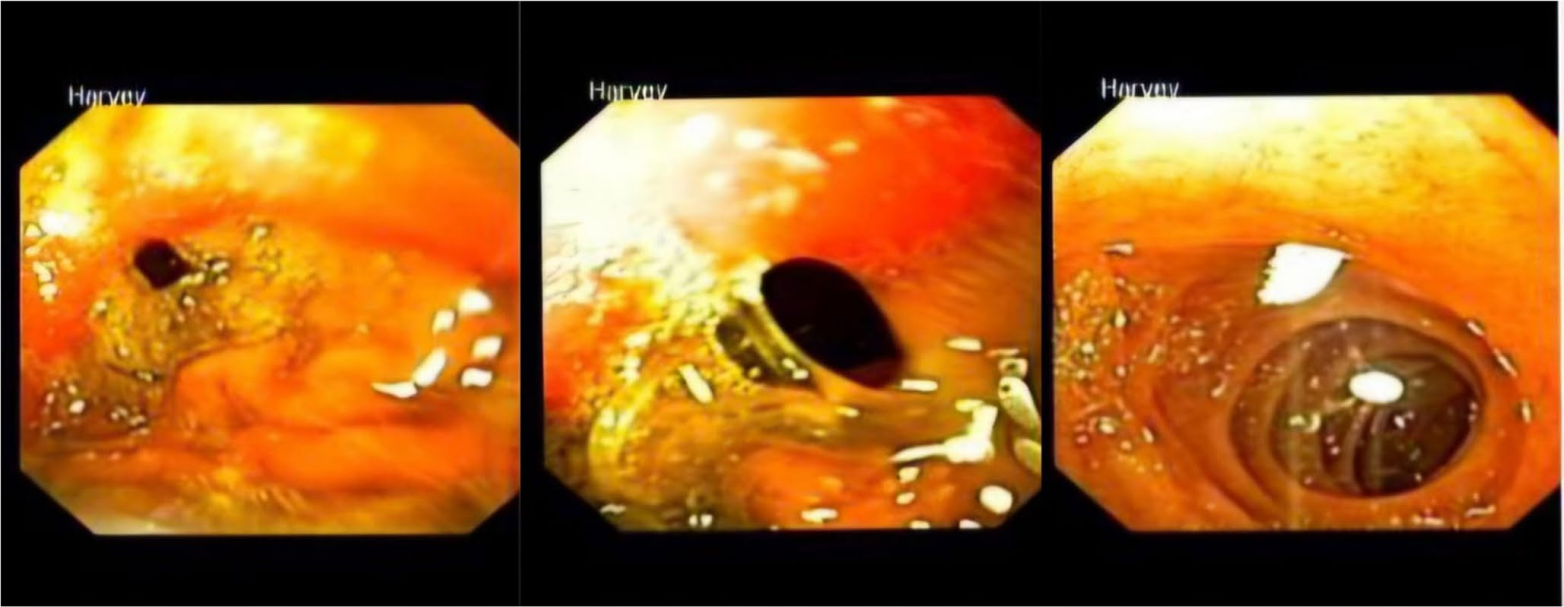
Upper GI endoscopy for a SASJ patient revealing severe biliary reflux and diffuse erythema of gastrojejunostomy and pyloric mucosa

Diagnosis is based on clinical presentation supported by endoscopic findings (illustrated in Fig. 1) and other objective assessments [8, 9]. While most cases respond to medical therapy, a subset remains refractory and requires surgical intervention [10–12].

Revisions include sleeve gastrectomy, which restores restrictive anatomy without diversion, and conversion to Roux-en-Y gastric bypass (RYGB), which diverts bile away from the gastric pouch [13–15]. However, comparative evidence in the setting of SASJ remains limited.

In patients with refractory biliary reflux following SASJ, this study compares the two revisional approaches.

## Materials and Methods

Between September 2021 and March 2025, several tertiary bariatric centers participated in this prospective comparative study. We included adult patients who needed revisional surgery and had persistent biliary reflux after Single Anastomosis Sleeve Jejunal (SASJ) bypass.

Bile pooling, gastritis, and mucosal damage are examples of endoscopically confirmed persistent symptoms of refractory biliary reflux. Patients were divided into two groups according to the type of revisional procedure they underwent: sleeve gastrectomy or conversion to Roux-en-Y gastric bypass (RYGB). The term “reversal” refers to sleeve revision rather than restoration of normal anatomy.

There were 40 patients in all (18 sleeve revisions and 22 RYGB). Procedure selection was guided by predefined anatomical and clinical criteria, including sleeve morphology, symptom severity, GERD presence, and endoscopic findings. Treatment allocation was anatomy-driven and based on predefined clinical and endoscopic criteria; therefore, randomization was not considered clinically appropriate in selected anatomical scenarios. Sleeve configuration was assessed primarily by upper gastrointestinal endoscopy and selectively by computed tomography when required. Patients with unfavorable anatomy were directed toward RYGB, while sleeve revision was reserved for those with preserved anatomy.

Inclusion criteria consisted of adults with persistent reflux despite at least six months of optimized medical therapy. Patients with incomplete follow-up, alternative procedures, or contraindications were excluded.

All operations were performed laparoscopically. RYGB involved pouch creation and Roux limb construction for bile diversion, whereas sleeve revision included division of the gastrojejunal anastomosis without bypass.

At one, three, six, and twelve months, follow-up was carried out. Improvement in reflux was the main result; complications, weight fluctuations, GERD progression, operative parameters, and readmission were the secondary outcomes.

Using SPSS version 25, statistical analysis was carried out with a significance level of *p* < 0.05.

## Results

Age differences between the two groups were not statistically significant (36.6 ± 9.35 years in Group A vs. 36.7 ± 8.72 years in Group B; *p* = 0.973). Likewise, there was no significant difference in baseline body mass index (35.29 ± 8.2 kg/m² vs. 33.26 ± 9.1 kg/m²; *p* = 0.463). Group A had a higher percentage of female patients (72.7%) than Group B (61.1%), but this difference was not statistically significant (*p* = 0.435). Diabetes mellitus, hypertension, hyperlipidemia, obstructive sleep apnea, osteoarthritis, gastroesophageal reflux disease, and marginal ulcers were among the preoperative comorbid conditions that were similar in both groups (all *p* > 0.05) (Table 1).

**Table 1:** Comparing the preoperative and demographic features of the patients in groups A and B

Preoperative data	Group A (RYGB)	Group B (Sleeve)	Test value	*P*-value	Sig.
	No. = 22	No. = 18			
Age					
Mean ± SD	36.6 ± 9.35	36.7 ± 8.72	0.035•	0.973	NS
Range	25–43	25–40			
**Sex**					
Female	16 (72.7%)	11 (61.1%)	0.609*	0.435	NS
Male	6 (27.3%)	7 (38.9%)			
**Duation (months)**					
Mean ± SD	6.2 ± 1.3	6.1 ± 1.7	−0.211•	0.834	NS
Range	5–7	5–7			
**6 months post SASI co-morbidities**					
Bile reflux	22 (100.0%)	18 (100.0%)	–	–	–
DM	3 (13.6%)	2 (11.1%)	0.058*	0.810	NS
HTN	2 (9.1%)	2 (11.1%)	0.045*	0.832	NS
Hyperlipidemia	2 (9.1%)	1 (5.6%)	0.178*	0.673	NS
OSA	1 (4.5%)	1 (5.6%)	0.021*	0.885	NS
OA	1 (4.5%)	1 (5.6%)	0.021*	0.885	NS
GERD	2 (9.1%)	1 (5.6%)	0.178*	0.673	NS
M.U	3 (13.6%)	1 (5.6%)	0.718*	0.397	NS
**Preoperative weight (kg)**					
Mean ± SD	97.6 ± 10.88	95.3 ± 9.24	−0.711•	0.482	NS
Range	83–105	80–102			
**Preoperative height (cm)**					
Mean ± SD	166.3 ± 8.9	169.4 ± 7.32	1.185•	0.243	NS
Range	160–178	165–180			
**Preoperative BMI (kg/m2)**					
Mean ± SD	35.29 ± 8.2	33.26 ± 9.1	−0.741•	0.463	NS
Range	28.3–42.1	25.7–41.5			

*P*-value > 0.05: Non-significant; P-value < 0.05: Significant; P-value < 0.01: Highly significant

DM: Diabetes mellitus; HTN: Hypertension; OSA: Obstructive sleep apnea;

OA: Osteoarthritis; GERD: Gastroesophageal reflux disease;

M.U: Marginal ulcer; BMI: Body mass index; SD: Standard deviation.

*: Chi-square test; •: Independent t-test

One patient in Group A needed to be converted to open surgery, whereas no such conversions occurred in Group B. Operative duration was significantly prolonged in Group A (173.5 ± 28.16 min) compared with Group B (96.71 ± 21.53 min; *p* < 0.001). 9.1% of patients in Group A had intraoperative findings, such as a large hiatal hernia requiring mesh repair or distal gastric stenosis requiring conversion to RYGB, which were not seen in Group B (*p* = 0.189) (Table 2).

**Table 2:** Comparing the intraoperative data of the patients under study between groups A and B

Intraoperative data	Group A (RYGB)	Group B (Sleeve)	Test value	*P*-value	Sig.
	No. = 22	No. = 18			
Revisional procedure					
Laparoscopic	21 (95.5%)	18 (100.0%)	0.839*	0.360	NS
Open	1 (4.5%)	0 (0.0%)			
**Operative time (min.)**					
Mean ± SD	173.5 ± 28.16	96.71 ± 21.53	−9.509•	< 0.001	HS
Range	120–200	75–120			
**Intraoperative findings/complications**					
Total number	2 (9.1%)	0 (0.0%)	1.722*	0.189	NS
* Large hiatus hernia	1 (4.5%)	0 (0.0%)	0.839*	0.360	NS
*Stenosis at distal gastric channel at level of incisura	1 (4.5%)	0 (0.0%)	0.839*	0.360	NS

*P*-value > 0.05: Non-significant; P-value < 0.05: Significant; P-value < 0.01: Highly significant

*: Chi-square test; •: Independent t-test

Group A had a significantly longer hospital stay (1.63 ± 0.52 days) than Group B (1.22 ± 0.67 days; *p* = 0.036). Two patients in each group experienced early postoperative complications. These included superficial surgical site infection (4.5%) and intraluminal bleeding (4.5%) in Group A, whereas contained leak (5.6%) and distal gastric stenosis (5.6%) were the complications in Group B. There was no significant difference in the overall complication rate between the groups (*p* = 0.832). One patient (4.5%) in Group A and two patients (11.1%) in Group B had readmissions within 30 days (*p* = 0.433) (Table 3).

**Table 3:** Comparing the early postoperative data of the patients under study between groups A and B

Early postoperative data	Group A (RYGB)	Group B (Sleeve)	Test value	*P*-value	Sig.
	No. = 22	No. = 18			
Hospital stay (days)					
Mean ± SD	1.63 ± 0.52	1.22 ± 0.67	−2.180•	0.036	S
Range	1.0–3.0	1–2.5			
**Postoperative complications**					
Total number	2 (9.1%)	2 (11.1%)	0.045*	0.832	NS
*Leak	0 (0.0%)	1 (5.6%)	1.254*	0.263	NS
*Intra luminal bleeding	1 (4.5%)	0 (0.0%)	0.839*	0.360	NS
*Surgical site infection	1 (4.5%)	0 (0.0%)	0.839*	0.360	NS
*Distal gastric stenosis	0 (0.0%)	1 (5.6%)	1.254*	0.263	NS
**Readmission in 30 days**	1 (4.5%)	2 (11.1%)	0.615*	0.433	NS

*P*-value > 0.05: Non-significant; P-value < 0.05: Significant; P-value < 0.01: Highly significant

*: Chi-square test; •: Independent t-test

At both 6 months and 1 year, biliary reflux was completely absent in Group A (0%), whereas persistent or recurrent reflux remained present in 27.8% of patients in Group B at both time points (*p* = 0.008). Regarding GERD, a reduction was observed in Group A from 9.1% preoperatively to 4.5% at 1 year, while Group B showed a marked increase from 5.6% to 38.9% (*p* = 0.007) (Table 4).

**Table 4:** Comparison of postoperative comorbidities among the patients in groups A and B following a year of surgery

Effect on comorbidities	Group A (RYGB)	Group B (Sleeve)	Test value	*P*-value	Sig.
	No. = 22	No. = 18			
Bile reflux					
1 month	8 (36.4%)	10 (55.6%)	1.473*	0.225	NS
3 months	2 (9.1%)	7 (38.9%)	5.041*	0.025	NS
6 months	0 (0.0%)	5 (27.8%)	6.984*	0.008	HS
1 year	0 (0.0%)	5 (27.8%)	6.984*	0.008	HS
**GERD after 1 year**	1 (4.5%)	7 (38.9%)	7.298*	0.007	HS
**M.U after 1 year**	0 (0.0%)	0 (0.0%)	–	–	–
**Comorbidities**					
DM	1 (4.5%)	4 (22.2%)	2.828*	0.093	NS
HTN	1 (4.5%)	3 (16.7%)	1.616*	0.204	NS
Hyperlipidemia	0 (0.0%)	2 (11.1%)	2.573*	0.109	NS
OSA	0 (0.0%)	1 (5.6%)	1.254*	0.263	NS
OA	0 (0.0%)	1 (5.6%)	1.254*	0.263	NS

*P*-value > 0.05: Non-significant; P-value < 0.05: Significant; P-value < 0.01: Highly significant

DM: Diabetes mellitus; HTN: Hypertension; OSA: Obstructive sleep apnea;

OA: Osteoarthritis; GERD: Gastroesophageal reflux disease;

M.U: Marginal ulcer.

*: Chi-square test; •: Independent t-test

Metabolic outcomes differed between groups. In Group A, the prevalence of diabetes mellitus declined from 13.6% to 4.5%, and hypertension decreased from 9.1% to 4.5%. Conversely, Group B demonstrated worsening metabolic parameters, with diabetes increasing from 11.1% to 22.2% and hypertension from 11.1% to 16.7% (Table 4).

Weight outcomes also showed distinct patterns. In Group A, BMI progressively decreased from 35.29 ± 8.2 kg/m² preoperatively to 31.61 ± 4.92 kg/m² at 6 months and 28.43 ± 5.28 kg/m² at 1 year (*p* < 0.001), with %EWL reaching 35.76% and 66.66%, respectively. In contrast, Group B experienced weight regain, with BMI increasing from 33.26 ± 9.1 kg/m² to 35.17 ± 5.31 kg/m² at 6 months and 36.82 ± 6.14 kg/m² at 1 year (Table 5).

**Table 5:** Comparing the effects of groups A and B on the patients’ weight loss following surgery

Effect on weight loss	Group A (RYGB)	Group B (Sleeve)	Test value	*P*-value	Sig.
	No. = 22	No. = 18			
Effect on weight loss					
BMI at 6 months	31.61 ± 4.92	35.17 ± 5.31	2.197•	0.034	S
EWL%	35.76% ± 5.27	-	-	-	-
BMI at 1 year	28.43 ± 5.28	36.82 ± 6.14	4.647•	< 0.001	HS
EWL%	66.66% ± 7.24	-	-	-	-

*P*-value > 0.05: Non-significant; P-value < 0.05: Significant; P-value < 0.01: Highly significant

*: Chi-square test; •: Independent t-test

## Discussion

After SASJ, the treatment of refractory biliary reflux is still difficult and has not received enough attention. This study demonstrates that conversion to RYGB provides more consistent improvement in reflux control, GERD outcomes, metabolic profile, and weight trajectory compared with sleeve revision.

Biliary reflux after loop-configured procedures is mainly due to retrograde movement of biliopancreatic secretions into the gastric lumen [16]. The anatomical configuration of RYGB effectively prevents this mechanism by separating biliary flow from the gastric pouch, explaining the high rates of symptom resolution observed [8, 13].

In contrast, sleeve revision does not eliminate the underlying reflux mechanism. The persistence of reflux can be attributed to factors such as pyloric relaxation and gastroduodenal motility, as no diversion pathway is created [17].

The differences in GERD outcomes align with existing evidence, as RYGB has consistently shown superior efficacy in GERD control, whereas sleeve procedures are associated with increased reflux risk [18–20].

Metabolic and weight outcomes also favored RYGB, likely due to combined restrictive and hormonal mechanisms, while sleeve revision provides limited metabolic benefit in this setting [21–25].

The study offers useful comparative data in a clinical scenario that is comparatively underreported, despite its limitations due to its non-randomized design, small sample size, and lack of sophisticated functional testing. Objective functional reflux assessment using Bilitec monitoring or hepatobiliary scintigraphy was not performed and remains a study limitation.

## Conclusion

Refractory biliary reflux after SASJ remains a challenging condition. Conversion to RYGB offers more reliable improvement in reflux and clinical outcomes compared with sleeve revision, despite longer operative time and hospital stay.

## Data Availability

the datasets generated and analyzed in this study are available from the corresponding author upon request.

## References

[1] Lee PC, Dixon J. Medical devices for the treatment of obesity. Nat Rev Gastroenterol Hepatol. 2017;14(9):553–64. 28698663 10.1038/nrgastro.2017.80

[2] Khalaf M, Hamed HJOS. Single-Anastomosis Sleeve Ileal (SASI) Bypass: Hopes and Concerns after a Two-Year Follow-up. Obes Surg. 2021;31(2):667–74. 32844276 10.1007/s11695-020-04945-y

[3] Yeung KTD, Penney N, Ashrafian L, et al. Does sleeve gastrectomy expose the distal esophagus to severe reflux? a systematic review and meta-analysis. Ann Surg. 2020;271(2):257–65. 30921053 10.1097/SLA.0000000000003275

[4] Mahdy T, Schou CJIOS. Efficacy of single anastomosis sleeve ileal (SASI) bypass for type-2 diabetic morbid obese patients: gastric bipartition, a novel metabolic surgery procedure—retrospective cohort study. Int J Surg. 2016;34:28–34. 27545956 10.1016/j.ijsu.2016.08.018

[5] Pazouki A, Kermansaravi M. Single anastomosis sleeve-jejunal bypass: a new method of bariatric/metabolic surgery. Obes Surg. 2019;29(11):3769–70. 31254211 10.1007/s11695-019-04016-x

[6] Sewefy AM, Atyia AM, Mohammed MM, et al. Single anastomosis sleeve jejunal (SAS-J) bypass as a treatment for morbid obesity: technique and review of 1986 cases and 6 years follow-up. Int J Surg. 2022;102:106662. 35568310 10.1016/j.ijsu.2022.106662

[7] Fontaine-Nicola A, Cambuli-Bianchi P, Sadiq KO, et al. Refractory bile reflux following one-anastomosis gastric bypass: A case report and literature review. Int J Surg Case Rep. 2025;133:111642. 40644983 10.1016/j.ijscr.2025.111642 PMC12275466

[8] Nimeri A, Al Shaban T, Maasher A. Conversion of one-anastomosis gastric bypass to Roux-en-Y gastric bypass for bile reflux gastritis after failed Braun jejunojejunostomy. Surg Obes Relat Dis. 2017;13(2):361–3. 27986575 10.1016/j.soard.2016.10.022

[9] Shenouda MM, Harb SE, Mikhail SA, et al. Bile gastritis following laparoscopic single anastomosis gastric bypass: significance of bilirubin in gastric aspirate. Obes Surg. 2018;28(2):389–95. 28849330 10.1007/s11695-017-2885-1

[10] Vosburg RW, Nimeri A, Azagury D, et al. ASMBS literature review on the treatment of marginal ulcers after metabolic and bariatric surgery. Surg Obes Relat Dis. 2025;21(1):1–8. 39516065 10.1016/j.soard.2024.10.003

[11] Saarinen T, Räsänen J, Salo J, et al. Bile reflux scintigraphy after mini-gastric bypass. Obes Surg. 2017;27(8):2083–9. 28214959 10.1007/s11695-017-2608-7

[12] Jedamzik J, Bichler C, Felsenreich DM, et al. Conversion from one-anastomosis gastric bypass to Roux-en-Y gastric bypass: when and why? Surg Obes Relat Dis. 2022;18:225–32. 34794865 10.1016/j.soard.2021.10.019

[13] Sargsyan N, Das B, Robb H. Outcomes of one-anastomosis gastric bypass conversion to Roux-en-Y gastric bypass for severe obesity: systematic review & meta-analysis. Obes Surg. 2024;34(3):976–84. 38244169 10.1007/s11695-023-07050-y PMC10899303

[14] Shah SS, Todkar JS. Roux-en-Y gastric bypass for intractable bile reflux after one-anastomosis gastric bypass: long-term outcomes. Obes Surg. 2020;30:1235–42.

[15] Aghajani E, Schou C, Gislason H, Nergaard BJ. Midterm outcomes after single anastomosis sleeve ileal (SASI) bypass. Surg Endosc. 2023;37:6220–7. 37171643 10.1007/s00464-023-10112-y PMC10338567

[16] Musella M, Milone M, Boschi S, et al. Rise of biliary reflux after one-anastomosis gastric bypass: pathophysiology & management. Surg Obes Relat Dis. 2019;15(8):1394–400.31285130

[17] Rebecchi F, Allaix ME, Patti MG, et al. GERD and laparoscopic sleeve gastrectomy: pathophysiology and prevention. World J Gastroenterol. 2014;20(39):14224–32.

[18] Frezza EE. Long-term GERD control after Roux-en-Y gastric bypass. Obes Surg. 2007;17(1):40–5.

[19] Stefanidis D, Kuwada TS, Gersin KS. The impact of RYGB on GERD: mechanisms and outcomes. Arch Surg. 2011;146(3):312–8.

[20] Qumseya B, Bitar A, Rachel A, et al. Systematic review and meta-analysis: LSG and GERD. Obes Surg. 2020;30(1):27–35.

[21] le Roux CW, Welbourn R, Werling M, et al. Gut hormone changes after bariatric surgery favor GLP-1–mediated metabolic improvement. Ann Surg. 2012;255(3):405–11.22330038

[22] Schauer PR, Kashyap SR, Wolski K, et al. Bariatric surgery vs medical therapy for diabetes: 3-year outcomes. N Engl J Med. 2014;370:2002–13. 24679060 10.1056/NEJMoa1401329 PMC5451259

[23] Moon RC, Teixeira AF, Snyder B, et al. Revisional bariatric surgery outcomes: RYGB vs SG. Surg Endosc. 2016;30(3):1227–32.26139483

[24] O’Brien PE, Dixon JB, Laurie C, et al. Ten-year outcomes after RYGB: durable weight-loss evidence. Ann Surg. 2019;269(2):273–81.

[25] Bhandari M, Fobi MA, Buchwald JN. Reversal procedures after bariatric surgery and their outcomes. Surg Obes Relat Dis. 2017;13(6):1017–27.

